# Emergence of direction- and orientation-selectivity and other complex structures from stochastic neuronal networks evolving under STDP

**DOI:** 10.1186/1471-2202-12-S1-P68

**Published:** 2011-07-18

**Authors:** Nana Arizumi, Todd Coleman, Lee DeVille

**Affiliations:** 1 Computer science, University of Illinois, Urbana-Champaign, IL 61801, USA; 2 Electrical Engineering, University of Illinois, Urbana-Champaign, IL 61801, USA; 3 Mathematics, University of Illinois, Urbana-Champaign, IL 61801, USA

## 

We consider neuronal network models with plasticity and randomness and show that complicated global structures can evolve even in the presence of simple local update rules. Our computational model generates several interesting features; e.g. orientation- and direction-selectivity when the inputs are arranged in a manner analogous to a visual field. Our model is a discrete-time Markov chain which contains multiple excitatory and inhibitory input neurons, and has as outputs stochastic leaky integrate-and-fire neurons; the system evolves through the plasticity of the synapses, updated according to a spike-timing dependent plasticity (STDP) rule.

We observe that the network is capable of rich properties (e.g. bifurcation, various forms of stability, etc) that depend on the statistics of the stimulus and the coupling parameters in the network. Since we are using a mechanism that can be easily modeled mathematically, we believe that this approach provides a well-positioned balance between neuro-biological relevance and theoretical tractability.

